# Hotspots and trends in multiple myeloma bone diseases: A bibliometric visualization analysis

**DOI:** 10.3389/fphar.2022.1003228

**Published:** 2022-10-14

**Authors:** Zhaomeng Hou, Ping Jiang, Shaoting Su, Honghai Zhou

**Affiliations:** ^1^ Guangxi University of Chinese Medicine, Nanning, China; ^2^ Yancheng TCM Hospital Affiliated to Nanjing University of Chinese Medicine, Yancheng, China; ^3^ Shanghai University of Traditional Chinese Medicine, Shanghai, China; ^4^ The First Affiliated Hospital of Guangxi University of Chinese Medicine, Nanning, China

**Keywords:** multiple myeloma bone disease, CiteSpace, bibliometrics, visualization analysis, Web of Science

## Abstract

**Objective:** This study aims to explore the research hotspots and trends of multiple myeloma bone disease in the past 20 years by bibliometric visualization analysis.

**Methods:** With the Web of Science Core Collection database as the data source, the relevant publications of multiple myeloma bone disease from 2002 to 2021 are retrieved. These data are analyzed using software CiteSpace 5.8.R3 and Scimago Graphica 1.0.24, together with the Online Analysis Platform of Literature Metrology.

**Results:** A total of 6,168 published research papers, including 4668 articles and 1500 review papers, are included in this study. Generally speaking, annual publications and citations are on the rise, especially in recent 2 years. The majority of these papers are published in the United States, with Mayo Clinic being the greatest contributor. The most productive journal and author are *Blood* and Terpos E, respectively, while the most frequently co-cited reference, author and journal are Rajkumar et al., 2014, *Lancet Oncol*, Kyle RA and *Blood*, respectively. The major research subject categories are oncology and hematology. The “disease diagnosis”, “prognosis evaluation”, “pathogenesis”, “imaging technology” and “targeted therapy” are recent research frontiers. The burst keywords “transplantation”, “progression”, “activation”, “lenalidomide”, “flow cytometry”, “drug resistance”, “management” and “mesenchymal stem cell” reflect the latest research hotspots.

**Conclusion:** This study reveals the research hotspots and trends of multiple myeloma bone disease through bibliometric visualization analysis, and provides a valuable reference for further research.

## Introduction

Multiple myeloma (MM) is a hematological malignancy characterized by malignant proliferation of plasma cells (Tomlinson, 2018; Joshua et al., 2019; Marina et al., 2020), accounting for about 1% of human malignancies and 10%–15% of hematological malignancies (Rajkumar, 2016). It is common among the middle-aged and the elderly, and has become the second highest incidence of hematological malignant tumors after non-Hodgkin’s lymphoma (Kumar et al., 2017; Wang et al., 2019; Von Suskil et al., 2021). Multiple myeloma bone disease (MBD) is the most common complication of MM, mainly due to the interaction between myeloma cells and bone marrow microenvironment, leading to increasing osteoclast activity and inhibition of osteoblast function, and resulting in bone metabolic imbalance and extensive osteolytic changes (Terpos et al., 2019; Zarghooni et al., 2019; Mateos et al., 2020). More than 80% of MM patients will develop MBD, which is characterized by bone pain, osteoporosis, hypercalcemia, pathological fracture, osteolytic destruction, spinal cord and nerve compression and a series of skeletal related effects (SRE), imposing a great impact on the prognosis and quality of life of the patients (Terpos et al., 2018; Visconti et al., 2021). At present, the main treatments to MBD are anti-MM therapy, targeted therapy, local radiotherapy, stem cell transplantation, surgical treatment and analgesia (Galán-Olleros et al., 2021). With further research and development, new treatment methods and drugs are emerging. It is also one of the important treatment methods to develop customized and more accurate medical care for MBD patients.

Bibliometric visualization analysis helps in figuring out the key paths and knowledge turning points of discipline development by measuring the literature in a specific field, and by drawing scientific knowledge maps to detect the frontiers of subject development (Dong et al., 2021). A variety of visual analysis software have been developed and used. Developed by Professor Chaomei Chen at Drexel University, CiteSpace has become one of the most widely used visualization software, and it is employed in many fields (Luo and Lin, 2021; Chen et al., 2022).

A great number of research papers have been published as the research goes on. However, to the best of our knowledge, so far there is no report on multiple myeloma bone disease from the perspective of bibliometrics. Using the software CiteSpace 5.8.R3 and Scimago Graphica 1.0.24, and the Bibliometrics Online Analysis Platform, along with the data sources of publications related to multiple myeloma bone disease included in the Web of Science Core Collection (WoSCC) database in recent 20 years, we have performed a bibliometric and visual analysis to identify research hotspots and trends in multiple myeloma bone disease, in an effort to contribute to the further research.

## Materials and methods

### Data source

Taking WoSCC database as the data source, all literature retrieval and data extraction were completed on 22 August 2022 to avoid deviation caused by database update. To improve retrieval accuracy, the subject entries were obtained from the standardized Medical Subject Headings (MeSH) list of the National Library of Medicine. The combination of subject headings and free words was used in retrieval, with the strategy: (TS = (multiple myeloma)) OR TS = (plasma cell myeloma)) OR TS = (myelomatosis)) OR TS = (Kahler disease)) AND TS = (bone disease)) OR TS = (myeloma bone disease)) AND DT = (Article OR Review)) AND LA = (English)) AND DOP = (2002-01-01/2021-12-31).

### Bibliometric analysis

The publications retrieved from the WoSCC database are exported in the plain text file in “full record and cited references” format and named in the form of “download_xxx.txt.” Then the downloaded documents are imported into the software CiteSpace 5.8.R3 to remove the duplicates. At the same time, all documents are downloaded in UTF-8 format with “full record and cited references” and imported into the Online Analysis Platform of Bibliometrics for analysis of cooperation between countries or regions. CiteSpace software parameter setting: Time Span chose from January 2002 to December 2021. Years Per Slice selected “2.” Term Source selected all by default. Nobe Types selected author, institution, country, keyword, category, reference, cited author, cited journal, respectively. Selection Criteria: top 50 per slice. Pruning chose pathfinder, pruning sliced networks and pruning the merged network, and other settings maintained the software default.

## Results and discussion

### Annual publications

**FIGURE 1:**
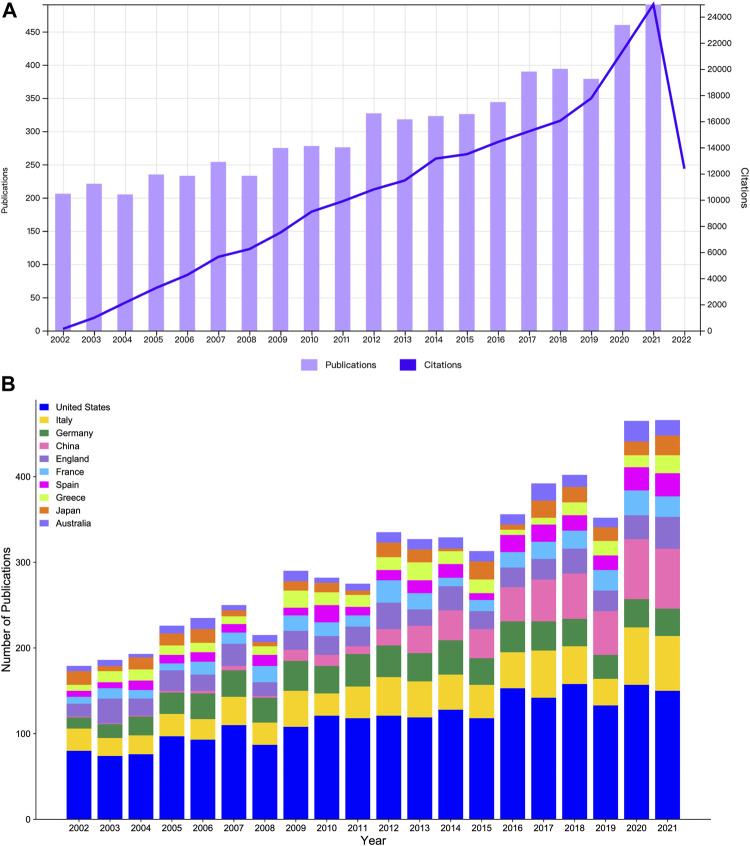
**(A)** The annual number of publications and citations on MBD between 2002 and 2021. **(B)** The number of publications in the top 10 countries/regions for MBD from 2002 to 2021.

Excluding other types of publications such as meeting abstracts, editorial materials, letters, corrections, book chapters, and retracted publications, a total of 6168 papers, including 4668 articles and 1500 review papers, are included in this study. The annual publications and citations are illustrated in Figure 1A. From 2002 to 2021, Annual publications and citations are on the rise in general, indicating that the research on MBD continued to attract researchers’ attention. Since 2020, the number of published papers and citations have been on rapid growing, indicating that the research of MBD has become more and more popular in recent years and attracted increasing attention of researchers. The top 10 countries/regions for publications are shown in Figure 1B. One can see from the figure that the United States is the leading country with the most publications, and the number of publications has increased steadily. Since 2016, the average annual number of publications has maintained around 150. There are few early publications in China. From 2002 to 2008, the average annual number of publications was about 2. Since 2009, its number increased rapidly, and in 2015, outnumbered that of Germany. Since 2018, it has exceeded that of Italy, being the second after the United States.

### Distribution of countries/regions and institutions

These 6,168 sample papers are published by 4,567 institutions in 101 countries/regions. The United States is the leading country with the most publications (2343, accounting for 37.99% of all publications, 125,048 total citations, 53.37 average citations per publication, 160 H-index, and 0.04 betweenness centrality), and the total citations and H-index also ranked the first. The H-index is a measure used to indicate the impact and productivity of a researcher based on how often his/her publications have been cited. The H-index of a publication is the largest number h such that at least h articles in that publication were cited at least h times each. For example, a journal with a h-index of 20 has published 20 articles that have been cited 20 or more times. It is used to evaluate the academic influence of journals, countries/regions, institutions or authors (Zhou et al., 2021).

**FIGURE 2:**
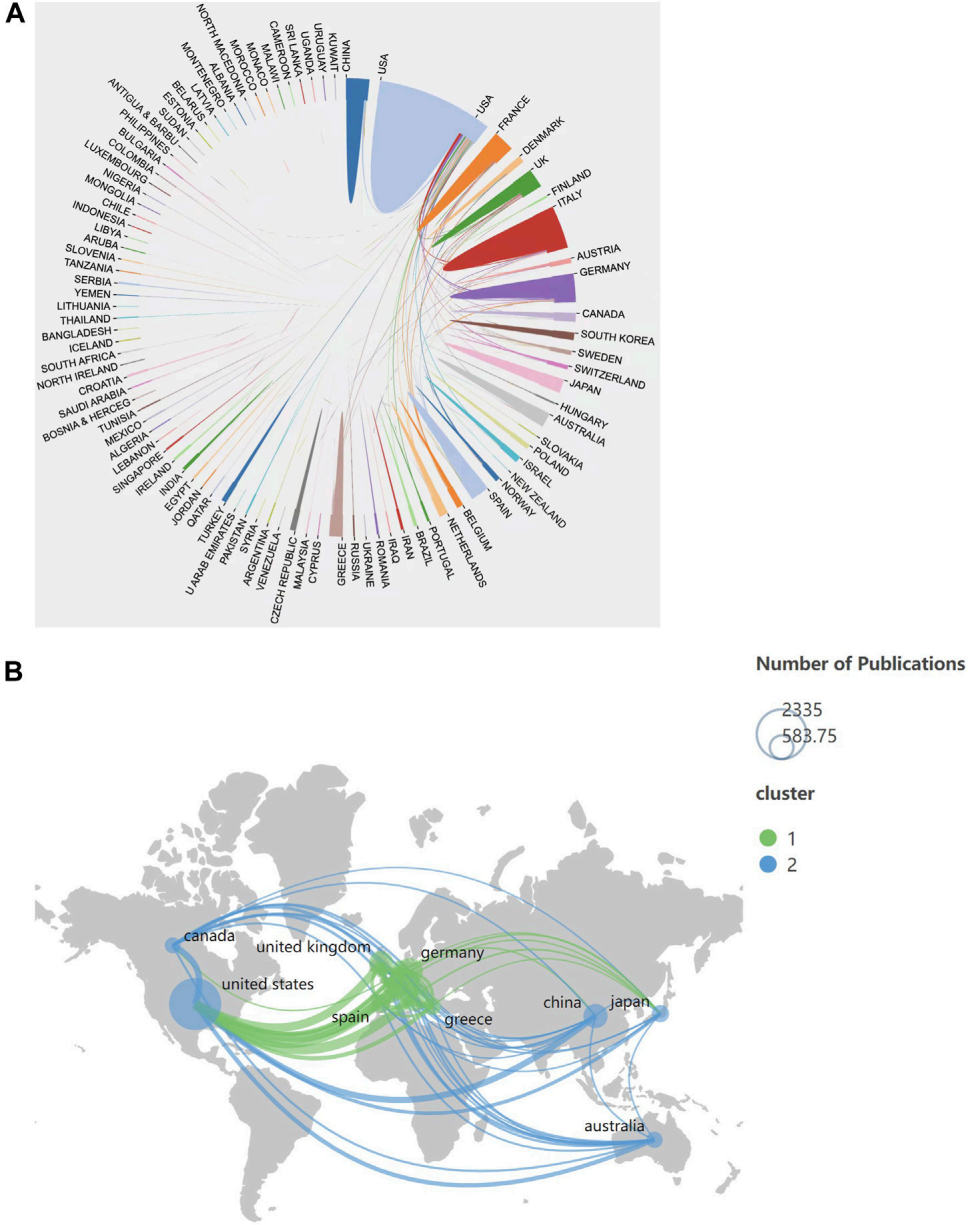
**(A)** Knowledge map of countries/regions cooperation network related to MBD. The map was produced by the Online Analysis Platform of Bibliometrics. **(B)** Geographical distribution of collaboration between countries/regions related to MBD. The graph was generated by Scimago Graphica 1.0.24 software. The size of the circles is proportional to the number of publications, the color of the circles represents the different clusters, and the thickness of the lines represents the strength of cooperation between countries/regions.

Following the United States, the second country is Italy (753, accounting for 12.21%, 40,479 total citations, 53.76 average citations per publication, 90 H-index, and 0.11 betweenness centrality), and followed by Germany (607, accounting for 9.84%, 33,545 total citations, 55.26 average citations per publication, 93 H-index, and 0.04 betweenness centrality) (Table 1). The top 10 countries/regions in publications are mostly from Europe or the United States, indicating that the main research contribution come from Europe and the United States. The betweenness centrality is an indicator of the importance of nodes in a network. The more important the nodes, the higher betweenness centrality as shown in the graph (Zhu et al., 2021). The top five countries/regions in terms of betweenness centrality are Finland (0.88), Jordan (0.75), Saudi Arabia (0.66), Thailand (0.62) and Estonia (0.60) (Supplementary Table S1). Although these countries/regions have a small number of publications, they cooperate closely with other countries/regions and play an important bridging role in this field. The United States cooperates closely with Italy and Germany, and also has extensive cooperation with other countries/regions. However, although China is a high-yield country, it has less cooperation with other countries/regions (Figures 2A,B). Therefore, international exchanges and cooperation should be strengthened to enhance its international influence in this research field.

**TABLE 1:** The top 10 publications of countries/regions related to MBD.

Rank	Countries/regions	Counts	Percentage	Total citations	Average citation per item	H-index	Centrality
1	United States	2,343	37.99	125,048	53.37	160	0.04
2	Italy	753	12.21	40,479	53.76	90	0.11
3	Germany	607	9.84	33,545	55.26	93	0.04
4	China	503	8.16	10,181	20.24	43	0.00
5	England	480	7.78	28,412	59.19	87	0.07
6	France	336	5.45	23,861	71.01	71	0.07
7	Spain	288	4.67	20,299	70.48	68	0.14
8	Greece	271	4.39	16,463	60.75	57	0.00
9	Japan	260	4.22	11,806	45.41	43	0.07
10	Australia	227	3.68	15,253	67.19	56	0.00

**FIGURE 3:**
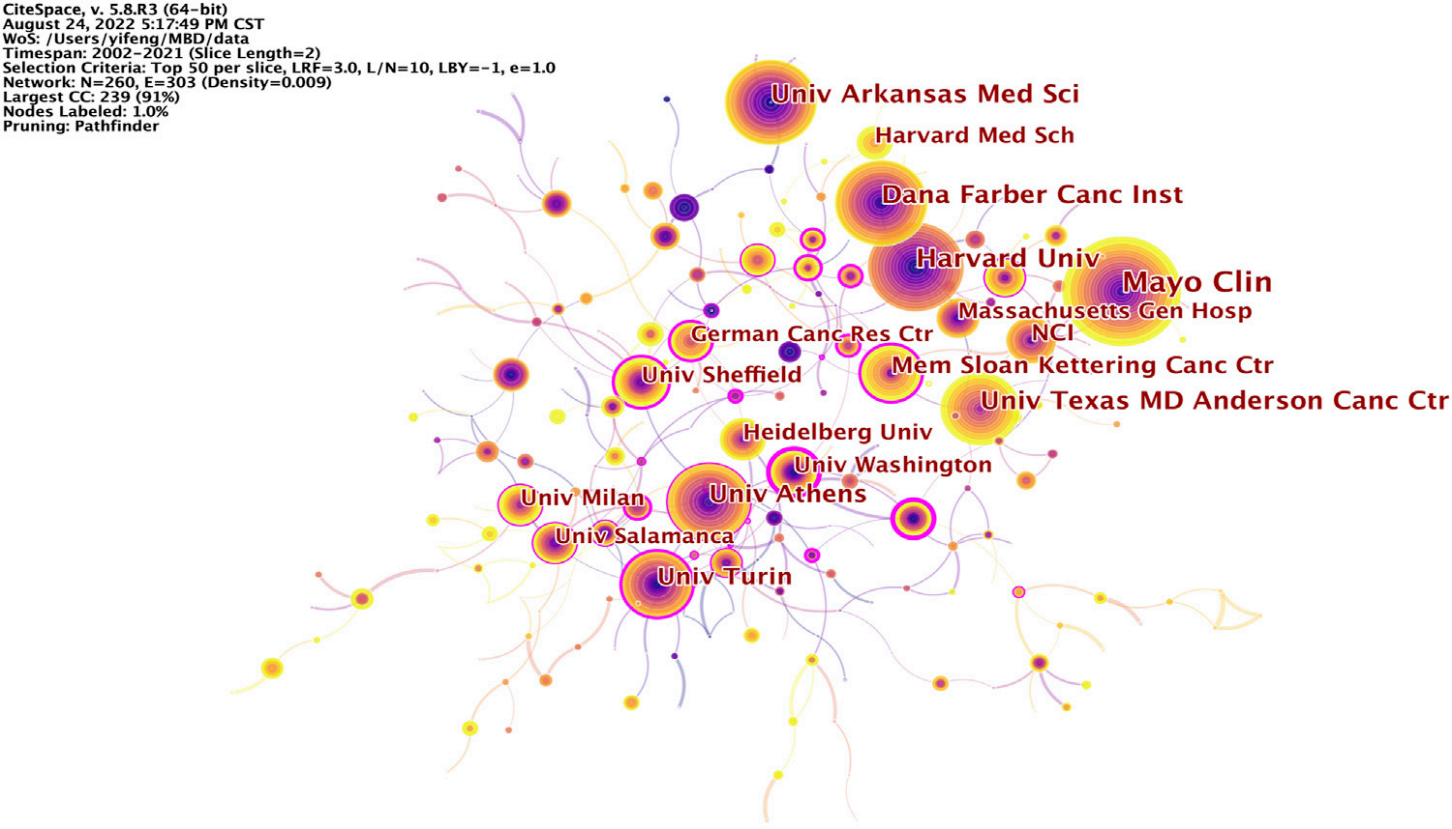
knowledge map of institutions cooperation network related to MBD. The size of the node represents the number of publications in the institutions, the color and thickness of the node represent the number of publications in different periods of time, and the warmer the color is, the closer the delivery time is. The line and its thickness indicate the cooperation and intensity of cooperation between institutions, while the color indicates the time of the first cooperation. The nodes with purple circles represent the key nodes whose betweenness centrality is not less than 0.1.

The knowledge map of institutions collaboration network consists of 260 nodes and 303 links, showing institutions with 60 or more publications (Figure 3). The top five institutions in publications are Mayo Clinic (254, 4.12%, 96.00 average citations per publication, 76 H-index, and 0.04 betweenness centrality), Harvard University (167, 2.71%, 96.09 average citations per publication, 90 H-index, and 0.07 betweenness centrality), University of Arkansas Medical Sciences (142, 2.30%, 77.90 average citations per publication, 59 H-index, and 0.03 betweenness centrality), Dana-Farber Cancer Institute (132, 2.14%, 111.13 average citations per publication, 80 H-index, and 0.08 betweenness centrality) and The University of Texas MD Anderson Cancer Center (127, 2.06%, 73.47 average citations per publication, 47 H-index, and 0.09 betweenness centrality) (Table 2). 80% of the top 10 institutions for publications are from the United States, indicating that research institutions in the United States have made outstanding contributions in this field. The top 10 institutions in publications have important academic prestige in various research fields. Among them, Mayo Clinic in the United States is one of the most influential medical institutions in the world and represents the world’s best medical research level, ranking first in the 2022 World’s best hospitals. Harvard University in the United States ranks first in the world in the 2022 Academic Ranking of World Universities. The top five institutions in terms of betweenness centrality are University of Washington (0.46), Fred Hutchinson Cancer Research Center (0.44), The Ohio State University (0.36), University of Turin (0.35) and Hospital Universitario de Salamanca (0.29) (Supplementary Table S1). These institutions are also all in Europe or the United States, acting as a bridge for the research results of different institutions in different periods. Therefore, through the analysis of the number of publications and the betweenness centrality of the institutions, one can see that the high-yield and high-centrality institutions are all from Europe or the United States, indicating that institutions in Europe and the United States are important research forces in this field. In contrast, although China has a large number of publications, there is no high-yield or high-centrality institution, indicating that China’s research level in this field is relatively poor and has not formed a research institution with core competitiveness. They should strengthen cooperation with major research institutions in China, Europe and the United States, and improve the overall strength of China’s research institutions.

**TABLE 2:** The top 10 publications of institutions related to MBD.

Rank	Institutions	Counts	Percentage	Average citation per item	H-index	Countries/regions	Centrality
1	Mayo Clin	254	4.12	96.00	76	United States	0.04
2	Harvard Univ	167	2.71	96.09	90	United States	0.07
3	Univ Arkansas Med Sci	142	2.30	77.90	59	United States	0.03
4	Dana Farber Canc Inst	132	2.14	111.13	80	United States	0.08
5	Univ Texas MD Anderson Canc Ctr	127	2.06	73.47	47	United States	0.09
6	Univ Athens	120	1.95	68.36	49	Greece	0.14
7	Mem Sloan Kettering Canc Ctr	104	1.69	66.34	33	United States	0.24
8	Univ Turin	96	1.56	122.22	53	Italy	0.35
9	National Cancer Institute (NCI)	80	1.30	67.00	38	United States	0.03
10	Massachusetts Gen Hosp	75	1.22	77.30	48	United States	0.02

### Analysis of journals and cited journals

A total of 1,235 journals published 6,168 publications. The top 10 most productive journals are listed in Table 3. Blood published the most papers (*n* = 222 (3.60%), with average citations per paper = 125.31, IF2021 = 25.476, H-index = 426, Q1), followed by British Journal of Haematology (*n* = 157 (2.55%), with average citations per paper = 46.84, IF2021 = 8.615, H-index = 172, Q1), Bone Marrow Transplantation (*n* = 153 (2.48%), with average citations per paper = 26.06, IF2021 = 5.174, H-index = 119, Q2), *Leukemia & Lymphoma* (*n* = 147 (2.38%), with average citations per paper = 16.90, IF2021 = 2.996, H-index = 82, Q3) and *Leukemia* (*n* = 125 (2.03%), with average citations per paper = 77.26, IF2021 = 12.883, H-index = 176, Q1). The average impact factor (IF) of the top 10 most productive journals is 8.883. Blood obtains the highest IF (25.476), with average citations per paper (125.31) and H-index (426), indicating that Blood is the most influential professional core journal in this field. 60% of the top 10 most productive journals are Q1, and 20% are Q2, indicating the high level of research in this field. The publishing countries/regions of the top 10 most productive journals are all from Europe or the United States, while the United States accounts for 30% and the United Kingdom accounts for 40%, indicating that journals from Europe and the United States have made important contributions in this field.

**TABLE 3:** The top 10 productive journals related to MBD.

Rank	Journal	Counts	Percentage	Average citation per item	H-index	IF(2021)	Quartile in category
1	Blood (United States)	222	3.60	125.31	426	25.476	Q1
2	Brit J Haematol (England)	157	2.55	46.84	172	8.615	Q1
3	Bone Marrow Transpl (England)	153	2.48	26.06	119	5.174	Q2
4	Leukemia Lymphoma (England)	147	2.38	16.90	82	2.996	Q3
5	Leukemia (England)	125	2.03	77.26	176	12.883	Q1
6	Biol Blood Marrow Tr (United States)	110	1.78	28.82	107	5.609	Q1
7	Eur J Haematol (Denmark)	98	1.59	21.74	76	3.674	Q3
8	Cancers (Switzerland)	96	1.56	7.70	53	6.575	Q1
9	Clin Cancer Res(United States)	86	1.39	57.55	292	13.801	Q1
10	Ann Hematol (Germany)	75	1.22	14.93	73	4.030	Q2

The knowledge map of co-cited journals network related to MBD consists of 88 nodes and 77 links, showing co-cited journals with citation frequency no less than 1,000 (Supplementary Figure S1). Journal co-citation refers to the phenomenon that two or more journals are cited by the same paper, revealing the correlation between various journals and disciplines, and obtaining the distribution of knowledge base in this field (Lu et al., 2021). Among them, *Blood* is in close co-citation relationships with British Journal of Haematology, New England Journal of Medicine, European Journal of Haematology, Haematologica and American Journal of Hematology, etc.; Journal of Clinical Oncology has strong co-citation relationships with New England Journal of Medicine, Leukemia, Bone Marrow Transplantation, Lancet, CANCER and Annals of Oncology, etc. The top 5 co-cited journals in terms of citation frequency are Blood (*n* = 4,818, IF2021 = 25.476, H-index = 426, Q1), British Journal of Haematology (*n* = 3,787, IF2021 = 8.615, H-index = 172, Q1), Journal of Clinical Oncology (*n* = 3,540, IF2021 = 50.717, H-index = 494, Q1), New England Journal of Medicine (*n* = 3,380, IF2021 = 176.079, H-index = 933, Q1) and *Leukemia* (*n* = 3,341, IF2021 = 12.883, H-index = 176, Q1) (Table 4). The top 5 co-cited journals for betweenness centrality are Journal of Clinical Oncology (*n* = 0.79, IF2021 = 50.717, H-index = 494, Q1), Cancer Research (*n* = 0.70, IF2021 = 13.312, H-index = 411, Q1), *Leukemia* (*n* = 0.69, IF2021 = 12.883, H-index = 176, Q1), Clinical Cancer Research (*n* = 0.63, IF2021 = 13.801, H-index = 292, Q1), and Proceedings of the National Academy of Sciences of the United States of America (*n* = 0.63, IF2021 = 12.779, H-index = 699, Q1) (Supplementary Table S2). *New England* Journal of Medicine is the co-cited journal with the highest IF (176.079) and H-index (933). Meanwhile, American journals account for the largest proportion of the top 10 co-cited journals in terms of citation frequency and betweenness centrality (60% and 80%, respectively), indicating that journals in the United States have an important academic prestige in the field and have received extensive attention. In addition, among the top 10 co-cited journals, in terms of citation frequency, the average IF is 32.771, 90% of which is Q1, and in terms of betweenness centrality, the average IF is 38.614, 90% of which is Q1, indicating that journals with high-IF are co-cited more frequently and play an important role in MBD research. Therefore, if a researcher is going to submit a research paper for publication, he may refer to articles published in the high-quality co-cited journals, and consider high-productivity journals first as well (Ke et al., 2020).

**TABLE 4:** The top 10 co-cited journals in terms of citation frequency related to MBD.

Rank	Co-cited Journal	Citation frequency	Centrality	H-index	IF(2021)	Quartile in category
1	Blood (United States)	4818	0.33	426	25.476	Q1
2	Brit J Haematol (England)	3787	0.04	172	8.615	Q1
3	J Clin Oncol (United States)	3540	0.79	494	50.717	Q1
4	New Engl J Med (United States)	3380	0.34	933	176.079	Q1
5	Leukemia (England)	3341	0.69	176	12.883	Q1
6	Clin Cancer Res(United States)	2326	0.63	292	13.801	Q1
7	Cancer Res(United States)	2177	0.70	411	13.312	Q1
8	Haematologica (Italy)	1964	0.00	120	11.047	Q1
9	Leukemia Lymphoma (England)	1849	0.00	82	2.996	Q3
10	P Natl Acad Sci USA (United States)	1766	0.63	699	12.779	Q1

### Analysis of authors and cited authors

**FIGURE 4:**
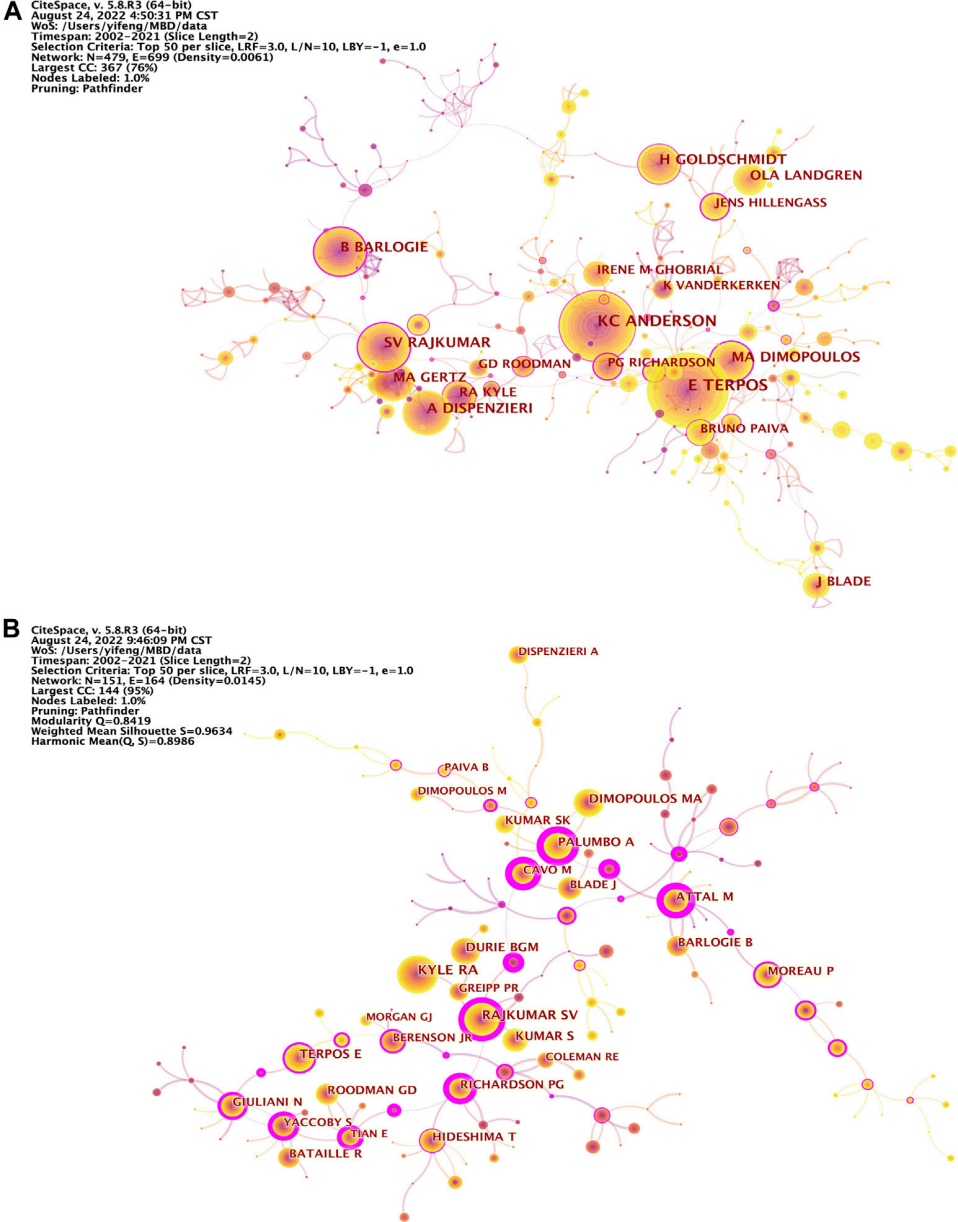
**(A)** Knowledge map of authors cooperation network related to MBD. The size of the node represents the number of publications in the authors, the color and thickness of the node represent the number of publications in different periods of time, and the warmer the color is, the closer the delivery time is. The line and its thickness indicate the cooperation and intensity of cooperation between authors, while the color indicates the time of the first cooperation. The nodes with purple circles represent the key nodes whose betweenness centrality is not less than 0.1. **(B)** Knowledge map of co-cited authors network related to MBD. The size of the node represents the number of co-citations, the color and thickness of the node represent the number of co-citations in different periods of time, and the warmer the color is, the closer the co-citation time is. The line and its thickness indicate the co-citation and co-citation strength between authors, while the color indicates the time of the first co-citation. The nodes with purple circles represent the key nodes whose betweenness centrality is not less than 0.1.

All these sampled papers about MBD are from a total of 26,131 authors. The Knowledge map of authors cooperation network consists of 479 nodes and 699 links, showing that authors with more than or equal to 40 publications (Figure 4A). The top 5 authors in terms of number of publications are Terpos E (142 papers, 2.30%, 72.13 average citations per paper, 50 H-index), Anderson KC (123 papers, 1.99%, 130.96 average citations per paper, 59 H-index), Dimopoulos MA (91 papers, 1.48%, 73.90 average citations per paper, 36 H-index), Rajkumar SV (89 papers, 1.44%, 172.82 average citations per paper, 51 H-index), Dispenzieri A (78 papers, 1.27%, 104.19 average citations per paper, 40 H-index) and Goldschmidt H (78 papers, 1.27%, 95.50 average citations per paper, 35 H-index) (Table 5). The top 5 authors for betweenness centrality are Dimopoulos MA (0.36, 73.90 average citations per paper, 36 H-index), Sezer O (0.31, 160.68 average citations per paper, 29 H-index), Richardson PG (0.23, 206.95 average citations per paper, 37 H-index), Hillengass J (0.22, 106.04 average citations per paper, 26 H-index), Rajkumar SV (0.20, 172.82 average citations per paper, 51 H-index), Barlogie B (0.20, 136.55 average citations per paper, 44 H-index) and Cavo M (0.20, 187.14 average citations per paper, 34 H-index) (Supplementary Table S3). Terpos E from the National and Kapodistrian University of Athens, Greece, is the author with the most publications, reflecting that he is academically highly influential with outstanding contributions in the field. His recent study showed that daratumumab improved bone turnover in relapsed/refractory Multiple Myeloma by inducing bone formation and reducing osteoblast inhibition (Terpos et al., 2022). On the other hand, Terpos E has active partnerships with Anderson KC, Dimopoulos MA, Rajkumar SV, Kyle RA, Roodman GD, Palumbo A, Cavo M, Durie BGM and Raje N. At the same time, this research field has also formed core research teams represented by Terpos E, Anderson KC, Dimopoulos MA, Rajkumar SV, Dispenzieri A and Goldschmidt H. In addition, further analysis shows that the top 10 authors with high-yield and high-centrality are all from Europe or the United States. In particular, the authors from the United States account for 70% and 60% respectively, indicating that researchers from Europe or the United States, especially the United States, have made important contributions and are academically highly influential in this field.

**TABLE 5:** The top 10 publications of authors related to MBD.

Rank	Author	Counts	Percentage	Average citation per item	H-index	Location	Centrality
1	Terpos E	142	2.30	72.13	50	Greece	0.08
2	Anderson KC	123	1.99	130.96	59	United States	0.19
3	Dimopoulos MA	91	1.48	73.90	36	Greece	0.36
4	Rajkumar SV	89	1.44	172.82	51	United States	0.20
5	Dispenzieri A	78	1.27	104.19	40	United States	0.01
6	Goldschmidt H	78	1.27	95.50	35	Germany	0.15
7	Barlogie B	77	1.25	136.55	44	United States	0.20
8	Gertz MA	68	1.10	57.68	38	United States	0.06
9	Kyle RA	66	1.07	148.56	41	United States	0.18
10	Landgren O	62	1.01	85.98	24	United States	0.04

The knowledge map of co-cited authors network related to MBD consists of 151 nodes and 164 links, showing co-cited authors with citation frequency no less than 300 (Figure 4B). Author co-citation refers to the phenomenon that two or more authors are cited by the same paper, which can reveal the academic community and by which one can figure out high-impact research groups in this field (Lin M. et al., 2021; Zhu et al., 2022). The top 5 co-cited authors in terms of citation frequency are Kyle RA (1,425 times), Rajkumar SV (1,238 times), Durie BGM (920 times), Terpos E (834 times) and Dimopoulos MA (804 times), while the top 5 co-cited authors for betweenness centrality are Palumbo A (1.06), Cavo M (1.03), Attal M (1.03), Rajkumar SV (0.97) and Richardson PG (0.90) (Table 6). Among them, Kyle RA from the Mayo Clinic in the United States is one of the top 10 authors in terms of publications and betweenness centrality, and is the co-cited author with the highest citation frequency at the same time. He published a paper in the New England Journal of Medicine titled: Multiple Myeloma, which systematically summarized the progress of multiple myeloma from the aspects of diagnosis, pathophysiology, treatment and management of complications (Kyle and Rajkumar, 2004). Palumbo A from the University of Torino in Italy is not only one of the top 10 co-cited authors with citation frequency, but also the co-cited author with the highest betweenness centrality. In his trial of 498 patients with relapsed or relapsed and refractory multiple myeloma, he showed that daratumumab combined with bortezomib and dexamethasone achieved significantly longer progression-free survival than bortezomib and dexamethasone alone (Palumbo et al., 2016). It is worth noting that the top 10 co-cited authors are still all from Europe or the United States in terms of citation frequency and betweenness centrality, especially the United States. Therefore, it is confirmed from many aspects that Europe and the United States, especially the latter, have a great academic research prestige in this field.

**TABLE 6:** The top 10 citation frequency and betweenness centrality of co-cited authors related to MBD.

Rank	Co-cited Author	Citation frequency	Location	Rank	Co-cited Author	Centrality	Location
1	Kyle RA	1425	United States	1	Palumbo A	1.06	Italy
2	Rajkumar SV	1238	United States	2	Cavo M	1.03	Italy
3	Durie BGM	920	United States	3	Attal M	1.03	France
4	Terpos E	834	Greece	4	Rajkumar SV	0.97	United States
5	Dimopoulos MA	804	Greece	5	Richardson PG	0.90	United States
6	Palumbo A	790	Italy	6	Alexanian R	0.89	United States
7	Kumar S	672	United States	7	Child JA	0.88	United Kingdom
8	Hideshima T	650	United States	8	Gahrton G	0.72	Sweden
9	Attal M	607	France	9	Tian E	0.70	United States
10	Moreau P	565	France	10	Zangari M	0.70	United States

### Analysis of cited references

**FIGURE 5:**
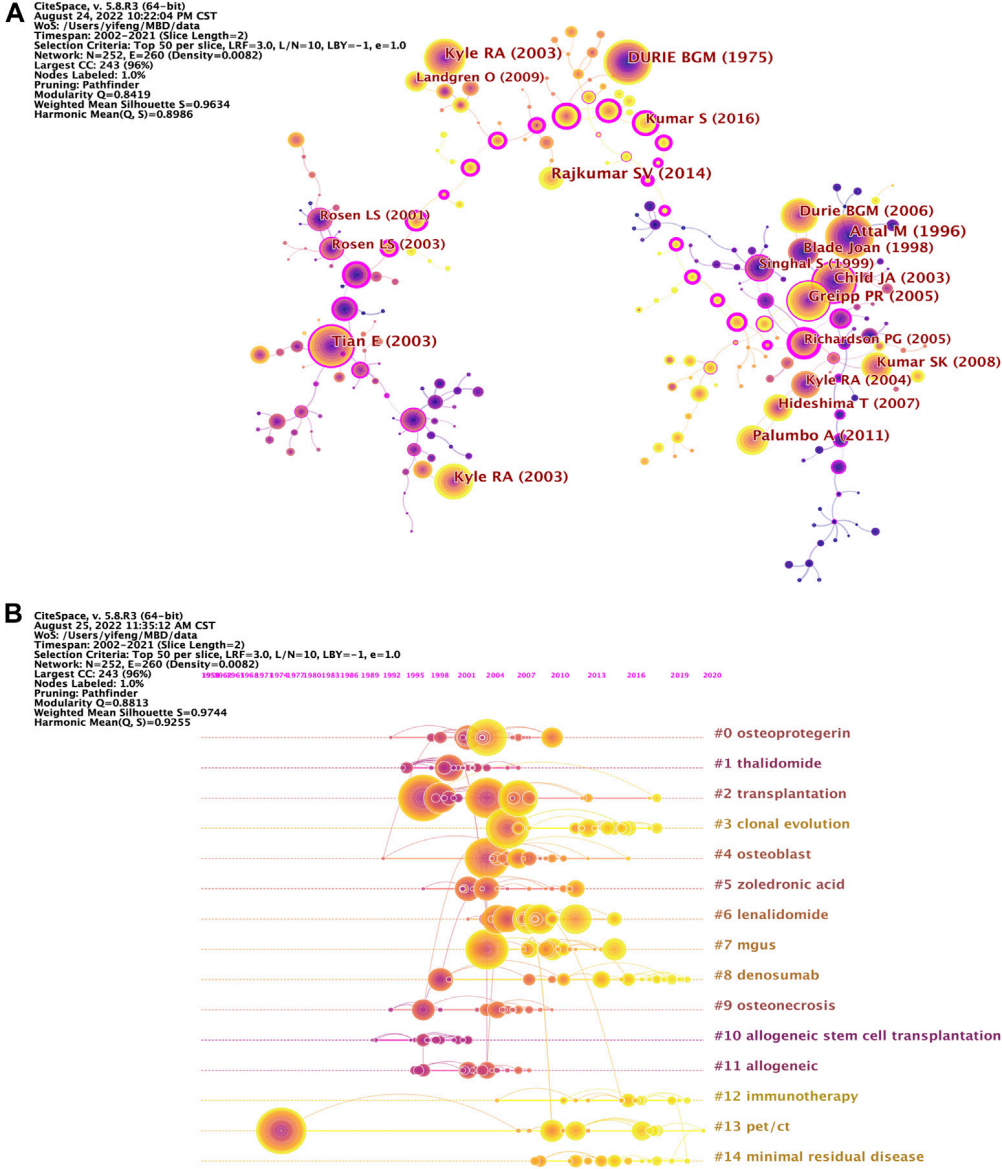
**(A)** Knowledge map of co-cited references network related to MBD. The size of the node represents the number of co-citations, the color and thickness of the node represent the number of co-citations in different periods of time, and the warmer the color is, the closer the co-citation time is. The line and its thickness indicate the co-citation and co-citation strength between references, while the color indicates the time of the first co-citation. The nodes with purple circles represent the key nodes whose betweenness centrality is not less than 0.1. **(B)** Timeline knowledge map of co-cited references related to MBD. The co-cited references of the same cluster are placed on the same horizontal line. The time the document co-citation is placed at the top of the view, and the farther to the right the closer the time is.

Co-cited references refer to the phenomenon that two or more publications are cited by the same paper. By analyzing the key nodes in the co-citation network, the knowledge base and research Frontier of the field can be explored, and papers with high academic influence and key roles can be found (Chen et al., 2019). The knowledge map of co-cited references network related to MBD consists of 252 nodes and 260 links, showing co-cited references with citation frequency no less than 150 (Figure 5A). The top 10 co-cited references according to citation frequency and betweenness centrality are listed in Tables 7, 8, respectively. The average IF of the journals that published these papers is 84.899 and 92.534, while the average H-index is 528.2 and 587.8, respectively. All these papers are Q1, indicating that these are high-quality papers with academically great influence in the field, all of which are the foundations of MBD research. Among them, the co-cited publication was published in Lancet Oncology by Rajkumar et al. (2014) in 2014 enjoys the highest citation frequency, which summarizes the International Myeloma Working Group consensus updated the disease definition of multiple myeloma. The second co-cited paper was published by Attal et al. (1996) in New England Journal of Medicine in 1996. Their study demonstrates that high-dose therapy combined with bone marrow transplantation improves patient response, event-free survival and overall survival in a randomized study of 200 untreated myeloma patients under the age of 65. In 1975, Durie and Salmon (1975) published the third co-cited study in *Cancer.* The authors developed a clinical staging system for multiple myeloma. British Journal of Haematology published the fourth co-cited publication by Kyle (2003) in 2003. This study reports the classification criteria of multiple myeloma, which helps evaluate the available prognostic factors to better define the prognosis of multiple myeloma. The fifth co-cited paper was published by Tian et al. (2003) in New England Journal of Medicine in 2003. The study finds that DKK1, produced by myeloma cells, is an inhibitor of osteoblastic differentiation and is associated with osteolytic bone lesions in patients with multiple myeloma. Through the analysis of the top 5 co-cited references with the citation frequency, one can find that the definition, diagnosis, staging, clinical presentation, treatment and complications management are mainly reviewed and studied, so these are the basis of MBD research.

**TABLE 7:** The top 10 co-cited references in terms of citation frequency related to MBD.

Rank	Co-cited reference	Author and publication year	Citation frequency	Journal IF (2021)	H-index	Quartile in category
1	International Myeloma Working Group updated criteria for the diagnosis of multiple myeloma	Rajkumar et al. (2014)	395	Lancet Oncol (IF:54.433)	274	Q1
2	A prospective, randomized trial of autologous bone marrow transplantation and chemotherapy in multiple myeloma. Intergroupe Français du Myélome	Attal et al. (1996)	382	New Engl J Med (IF:176.079)	933	Q1
3	Clinical staging system for multiple-myeloma-correlation of measured myeloma cell mass with presenting clinical features, response to treatment, and survival	Durie and Salmon, (1975)	355	Cancer (IF:6.921)	277	Q1
4	Criteria for the classification of monoclonal gammopathies, multiple myeloma and related disorders: a report of the International Myeloma Working Group	Kyle, (2003)	337	Brit J Haematol (IF:8.615)	172	Q1
5	The role of the Wnt-signaling antagonist DKK1 in the development of osteolytic lesions in multiple myeloma	Tian et al. (2003)	329	New Engl J Med (IF:176.079)	933	Q1
6	Multiple myeloma	Palumbo and Anderson, (2011)	309	New Engl J Med (IF:176.079)	933	Q1
7	International staging system for multiple myeloma	Greipp et al., (2005)	309	J Clin Oncol (IF:50.717)	494	Q1
8	Review of 1027 patients with newly diagnosed multiple myeloma	Kyle et al., (2003)	307	Mayo Clin Proc (IF:11.104)	157	Q1
9	International uniform response criteria for multiple myeloma	Durie et al., (2006)	281	Leukemia (IF:12.883)	176	Q1
10	High-dose chemotherapy with hematopoietic stem-cell rescue for multiple myeloma	Child et al., (2003)	277	New Engl J Med (IF:176.079)	933	Q1

**TABLE 8:** The top 10 co-cited references for betweenness centrality related to MBD.

Rank	Co-cited reference	Author and publication year	Centrality	Journal IF (2021)	H-index	Quartile in category
1	Bortezomib or high-dose dexamethasone for relapsed multiple myeloma	Richardson et al., (2005)	1.05	New Engl J Med (IF:176.079)	933	Q1
2	Minimal residual disease negativity using deep sequencing is a major prognostic factor in multiple myeloma	Perrot et al. (2018)	1.01	Blood (IF:25.476)	426	Q1
3	F18-fluorodeoxyglucose positron emission tomography in the context of other imaging techniques and prognostic factors in multiple myeloma	Bartel et al. (2009)	0.96	Blood (IF:25.476)	426	Q1
4	Prognostic relevance of 18-F FDG PET/CT in newly diagnosed multiple myeloma patients treated with up-front autologous transplantation	Zamagni et al. (2011)	0.95	Blood (IF:25.476)	426	Q1
5	Elotuzumab Therapy for Relapsed or Refractory Multiple Myeloma	Lonial et al. (2015)	0.95	New Engl J Med (IF:176.079)	933	Q1
6	Daratumumab, Lenalidomide, and Dexamethasone for Multiple Myeloma	Dimopoulos et al. (2016)	0.95	New Engl J Med (IF:176.079)	933	Q1
7	Magnetic resonance imaging in multiple myeloma: diagnostic and clinical implications	Walker et al. (2007)	0.95	J Clin Oncol (IF:50.717)	494	Q1
8	Prognostic significance of focal lesions in whole-body magnetic resonance imaging in patients with asymptomatic multiple myeloma	Hillengass et al. (2010)	0.94	J Clin Oncol (IF:50.717)	494	Q1
9	Daratumumab plus Bortezomib, Melphalan, and Prednisone for Untreated Myeloma	Mateos et al. (2019)	0.94	New Engl J Med (IF:176.079)	933	Q1
10	International Myeloma Working Group consensus criteria for response and minimal residual disease assessment in multiple myeloma	Kumar et al. (2016)	0.93	Lancet Oncol (IF:54.433)	274	Q1
11	Next Generation Flow for highly sensitive and standardized detection of minimal residual disease in multiple myeloma	Flores-Montero et al. (2017)	0.93	Leukemia (IF:12.883)	176	Q1
12	Bortezomib, thalidomide, and dexamethasone with or without daratumumab before and after autologous stem-cell transplantation for newly diagnosed multiple myeloma (CASSIOPEIA): a randomised, open-label, phase 3 study	Moreau et al. (2019)	0.93	Lancet (IF:202.731)	700	Q1
13	Measurable Residual Disease by Next-Generation Flow Cytometry in Multiple Myeloma	Paiva et al. (2020)	0.93	J Clin Oncol (IF:50.717)	494	Q1

The co-cited reference with the highest betweenness centrality was published by Richardson et al. (2005) in New England Journal of Medicine in 2005. This study finds that bortezomib is more advantageous than high-dose dexamethasone in the treatment of relapsed multiple myeloma. In 2018, the second co-cited paper for betweenness centrality was published in Blood by Perrot et al. (2018). Their research confirms the value of minimal residual disease status determined by next-generation sequencing as a prognostic biomarker for multiple myeloma. Bartel et al. (2009) published the third co-cited publication for betweenness centrality in Blood in 2009. The authors provide a theoretical basis for the hypothesis that patients with multiple myeloma who fail to achieve FDG suppression after induction therapy can improve myeloma survival with a change in treatment by examining three imaging techniques: metastatic bone survey, magnetic resonance imaging, and FDG-PET scanning. The fourth co-cited study for betweenness centrality was published in Blood by Zamagni et al. (2011) in 2011. It demonstrates that PET/CT is a reliable technique for predicting long-term prognosis of patients with autotransplantation at diagnosis and after autologous stem cell transplantation. The fifth co-cited paper for betweenness centrality was published by Lonial et al. (2015) in New England Journal of Medicine in 2015. This study reveals that elotuzumab combined with lenalidomide and dexamethasone in the treatment of relapsed or refractory multiple myeloma reduce the risk of disease progression or death by 30% compared with lenalidomide plus dexamethasone. In 2016, the sixth co-cited paper for betweenness centrality was published in New England Journal of Medicine by Dimopoulos et al. (2016). This study confirms that daratumumab in combination with lenalidomide and dexamethasone significantly prolong progression-free survival in patients with relapsed or refractory multiple myeloma. Walker et al. (2007) published the seventh co-cited publication for betweenness centrality in Journal of Clinical Oncology in 2007. This study suggests that MRI should also be routinely used for the staging, prognosis and response evaluation of myeloma in addition to the metabolic bone survey. According to the analysis of the top 7 co-cited references of betweenness centrality, the main research topics are detection or evaluation of the prognosis of MM through imaging techniques such as PET/CT and MRI or tools such as biomarkers, and to treat MM with combination therapy. This reflects, to some extent, the research Frontier in this field. There are 15 clusters in the timeline map of co-cited references, which reflects the time span of co-cited references and the rise, prosperity and decline of a specific cluster research (Figure 5B). It can find from the figure that # 3 clonal evolution, # 8 denosumab, # 12 immunotherapy, # 13 PET/CT, # 14 minimal residual disease are research hotspots in recent years.

### References with citation burstness

**FIGURE 6:**
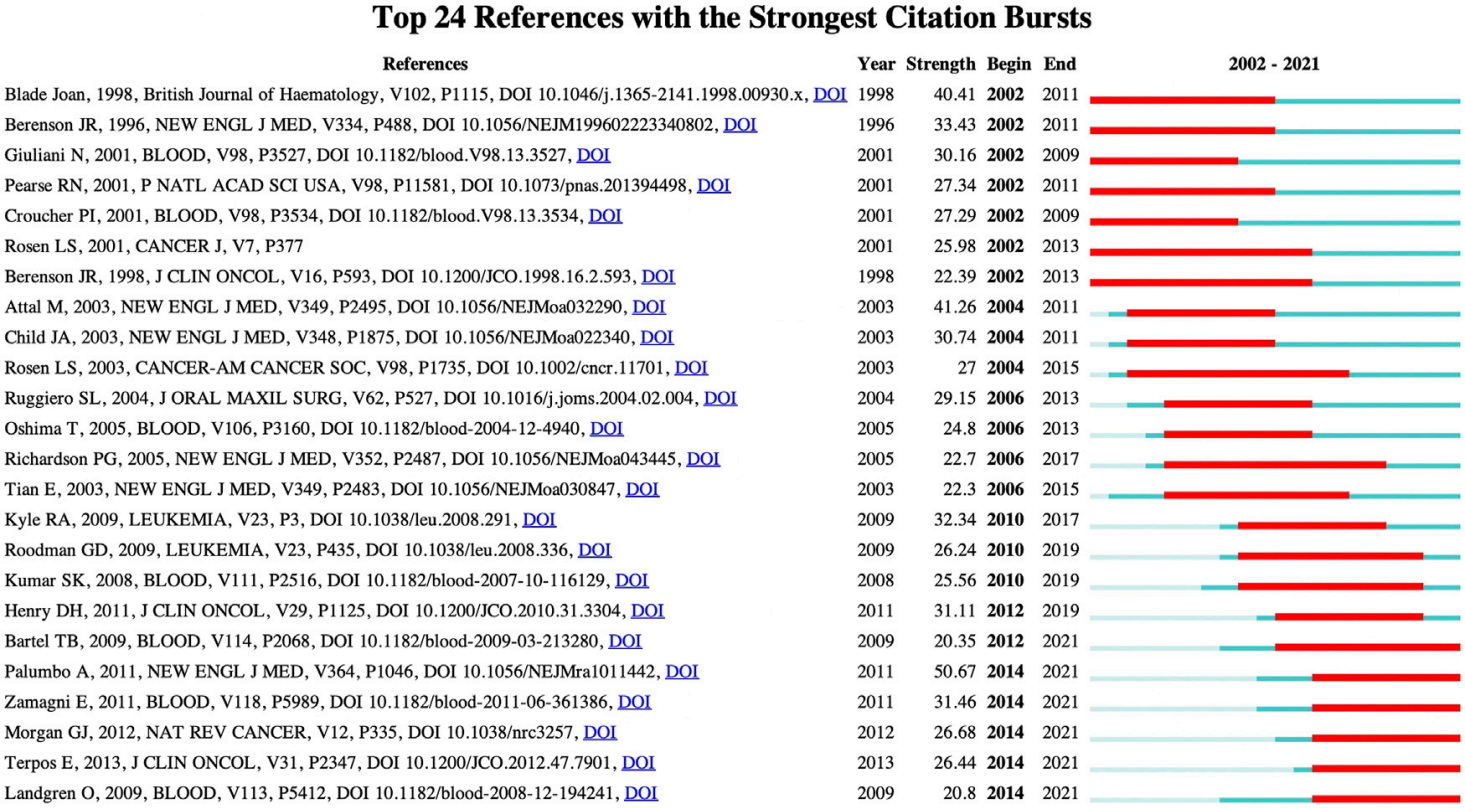
Top 24 references with the strongest citation bursts. The blue line indicates the year of the outbreak, and the red line indicates the time from the beginning to the end of the co-cited reference.

Citation burstness refers to citation surge of some paper in a short period of time. By analyzing the references with citation burst, the research trend in this field can be predicted (Lu et al., 2020). The top 24 references with the strongest citation bursts are detected by setting the minimum duration of the burst to 7 years (Figure 6). The blue line indicates the year of the outbreak, while the red line represents the time from the beginning to the end of the co-cited reference. “Strength” represents the burst strength. The larger the value, the higher the strength and the greater the influence of the publication (Zhang T. et al., 2022). Among the top 24 references with the strongest citation bursts, the citation burst of six references ended in 2021. Therefore, they reflect the latest research trends in MBD research and will be further discussed. The first reference with the greatest burst strength was published by Palumbo and Anderson (2011) in New England Journal of Medicine in 2011. This review summarizes the medical progress of multiple myeloma from the aspects of biology, clinical presentation, diagnosis, staging, treatment and management of adverse events related to therapy*.* In 2011, the paper with the second highest burst strength of the six publications with the citation burst was published in blood by Zamagni et al. (2011). Their study shows that PET/CT is a reliable technique for predicting long-term prognosis of patients with autotransplantation at diagnosis and after autologous stem cell transplantation. Morgan et al. (2012) published the study with the third highest citation burst in Nature Reviews Cancer in 2012. This review systematically introduces the genetic architecture of multiple myeloma. The publication with the fourth highest citation burst was published by Terpos et al. (2013) in Journal of Clinical Oncology in 2013. This study recommends use of bisphosphonates, kyphoplasty, or low-dose radiation for the treatment of multiple myeloma-related bone disease. Blood published the paper with the fifth highest citation burst by Landgren et al. (2009) in 2009. This study shows that the asymptomatic monoclonal gammopathy of undetermined significance (MGUS) stage always precedes MM. Finally, Bartel et al. (2009) published the study with the sixth highest citation burst in blood in 2009. The authors provide a theoretical basis for the hypothesis that patients with multiple myeloma who fail to achieve FDG suppression after induction therapy can improve myeloma survival with a change in treatment by examining three imaging techniques: metastatic bone survey, magnetic resonance imaging, and FDG-PET scanning. Through the analysis of these six publications, one can find that disease diagnosis, prognosis evaluation, pathogenesis, targeted therapy and imaging technology are the current research trends in the field of MBD research.

### Analysis of subject categories

**FIGURE 7:**
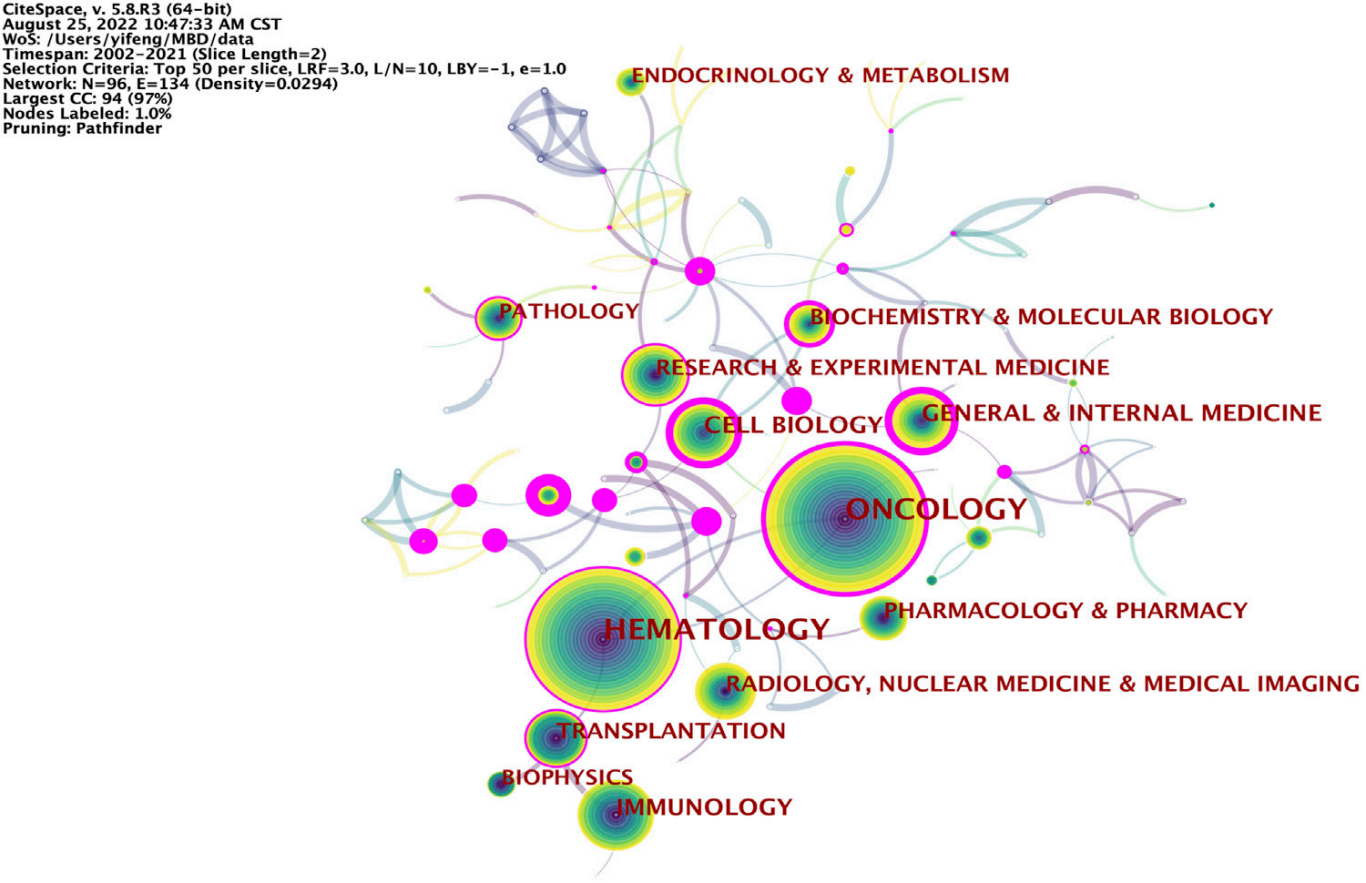
Knowledge map of category co-occurrence network related to MBD. The size of the node represents the frequency of categories, the color and thickness of the node represent the frequency of categories in different periods of time, and the warmer the color is, the closer the appearance time is. The line and its thickness indicate the co-occurrence and intensity of co-occurrence between categories, while the color indicates the time of the first co-occurrence. The nodes with purple circles represent the key nodes whose betweenness centrality is not less than 0.1.

**FIGURE 8:**
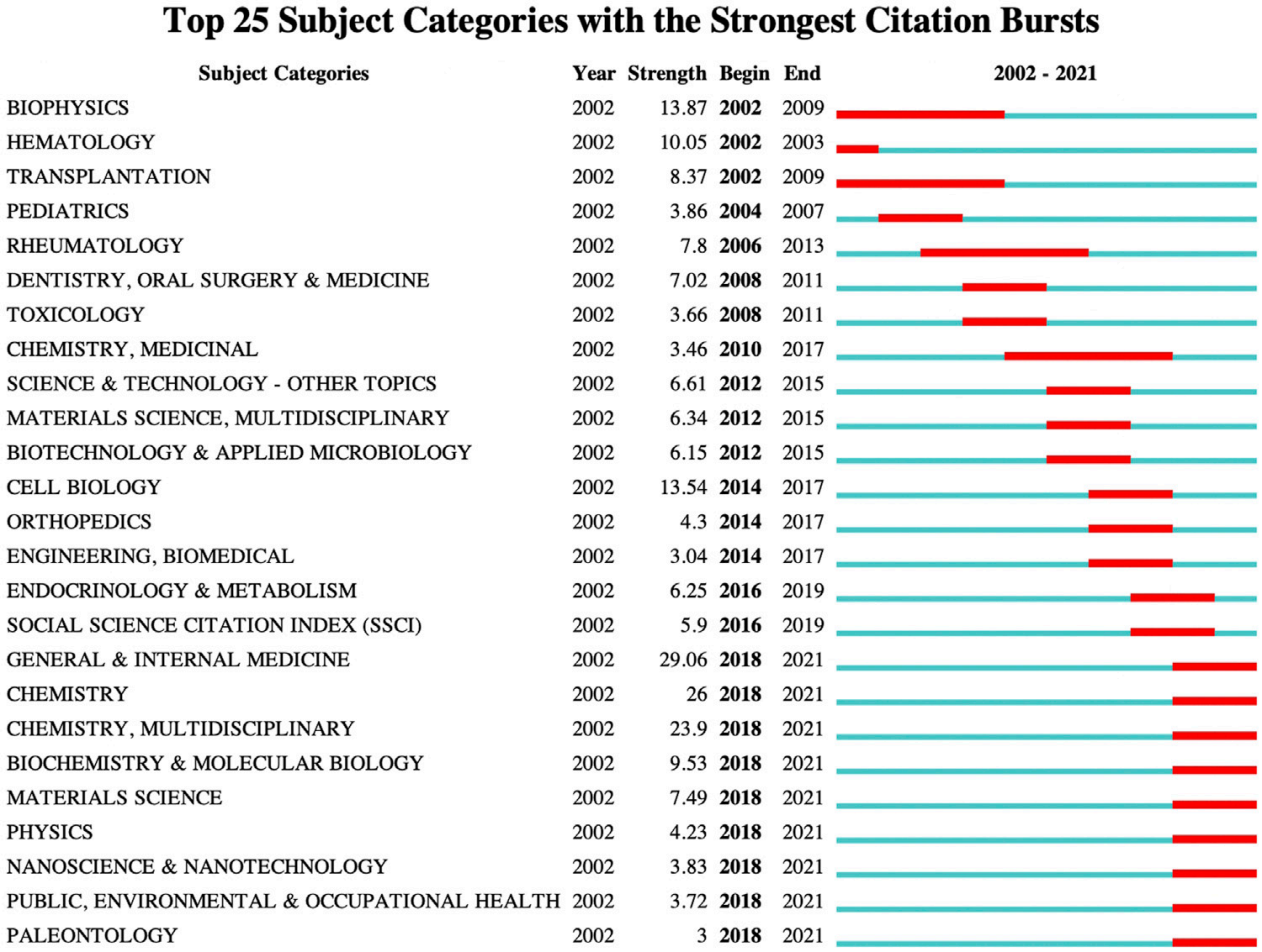
Top 25 subject categories with the strongest citation bursts. The blue line indicates the year of the outbreak, and the red line indicates the time from the beginning to the end of the subject categories.

The knowledge map of category co-occurrence network related to MBD consists of 96 nodes and 134 links. Shown in Figure 7 are subject categories with a frequency no less than 150. Among them, the top 5 subject categories for frequency of occurrence are Oncology (2098), Hematology (1996), Immunology (476), General Internal Medicine (394) and Cell Biology (342) (Supplementary Table S4). These are the main research subjects in this field, and have been studied extensively. The top 5 subject categories for betweenness centrality are Public Environmental Occupational Health (1.12), Environmental Sciences Ecology (1.09), Science Technology Other Topics (0.93). Nanoscience Nanotechnology (0.93) and Cell Tissue Engineering (0.88) (Supplementary Table S4). These disciplines are closely connected with other disciplines and play a bridging role in the interdisciplinary research. The top 25 subject categories with the strongest citation bursts are detected by setting the minimum duration of the burst to 2 years (Figure 8). Among the top 25 subject categories with the strongest citation bursts, the citation burst of nine subject categories ended in 2021. Therefore, General Internal Medicine, Chemistry Multidisciplinary, Biochemistry Molecular Biology, Materials Science, Physics, Nanoscience Nanotechnology, Public Environmental Occupational Health and Paleontology are hot subject categories in this research field.

### Analysis of keywords

**FIGURE 9:**
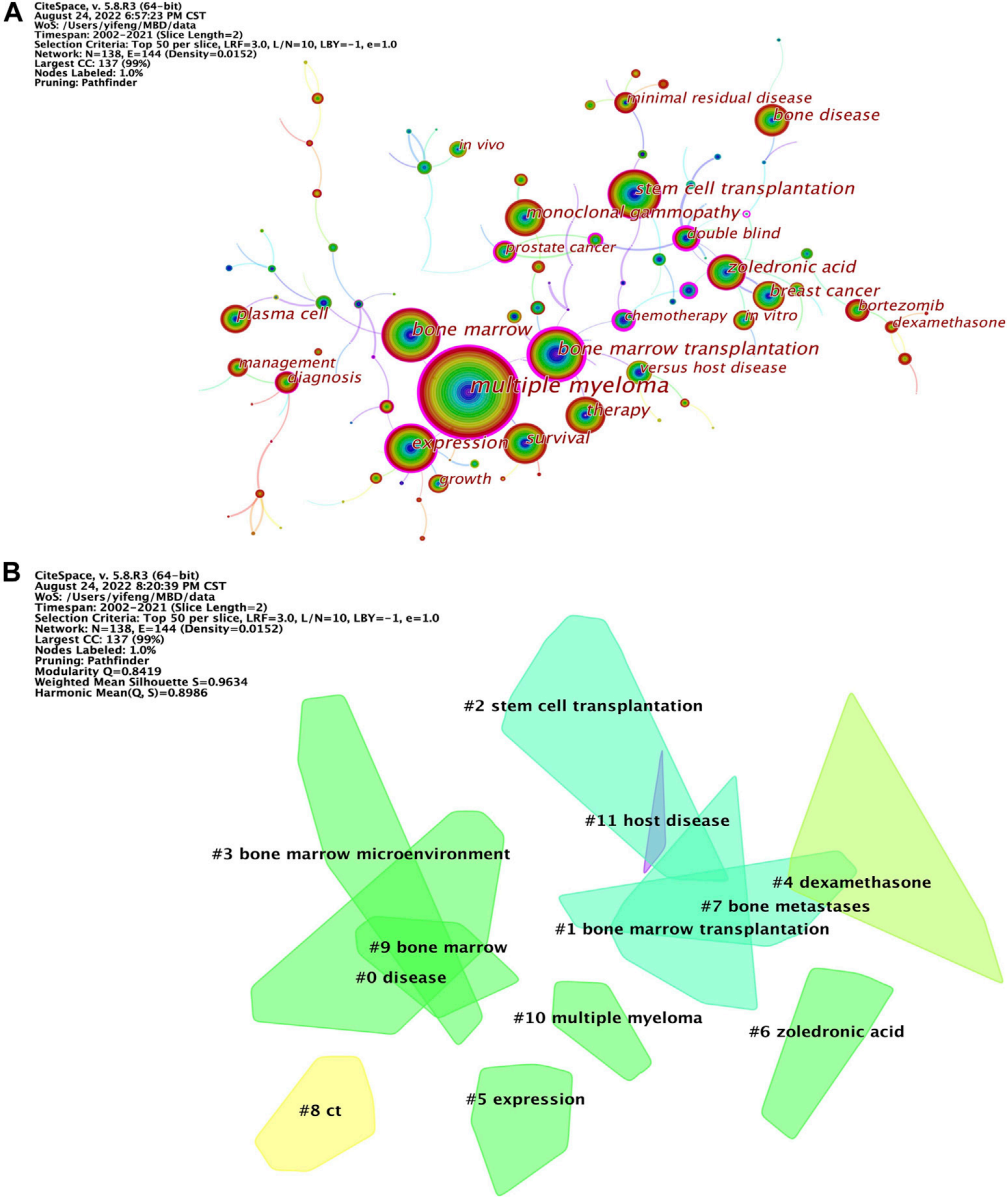
**(A)** Knowledge map of keyword co-occurrence network related to MBD. The size of the node represents the frequency of keywords, the color and thickness of the node represent the frequency of keywords in different periods of time, and the warmer the color is, the closer the appearance time is. The line and its thickness indicate the co-occurrence and intensity of co-occurrence between keywords, while the color indicates the time of the first co-occurrence. The nodes with purple circles represent the key nodes whose betweenness centrality is not less than 0.1. **(B)** Knowledge map of keyword clustering network related to MBD. Each color block represents a cluster. The smaller the number is, the larger the scale of the cluster is and the more keywords are contained in the cluster.

**FIGURE 10:**
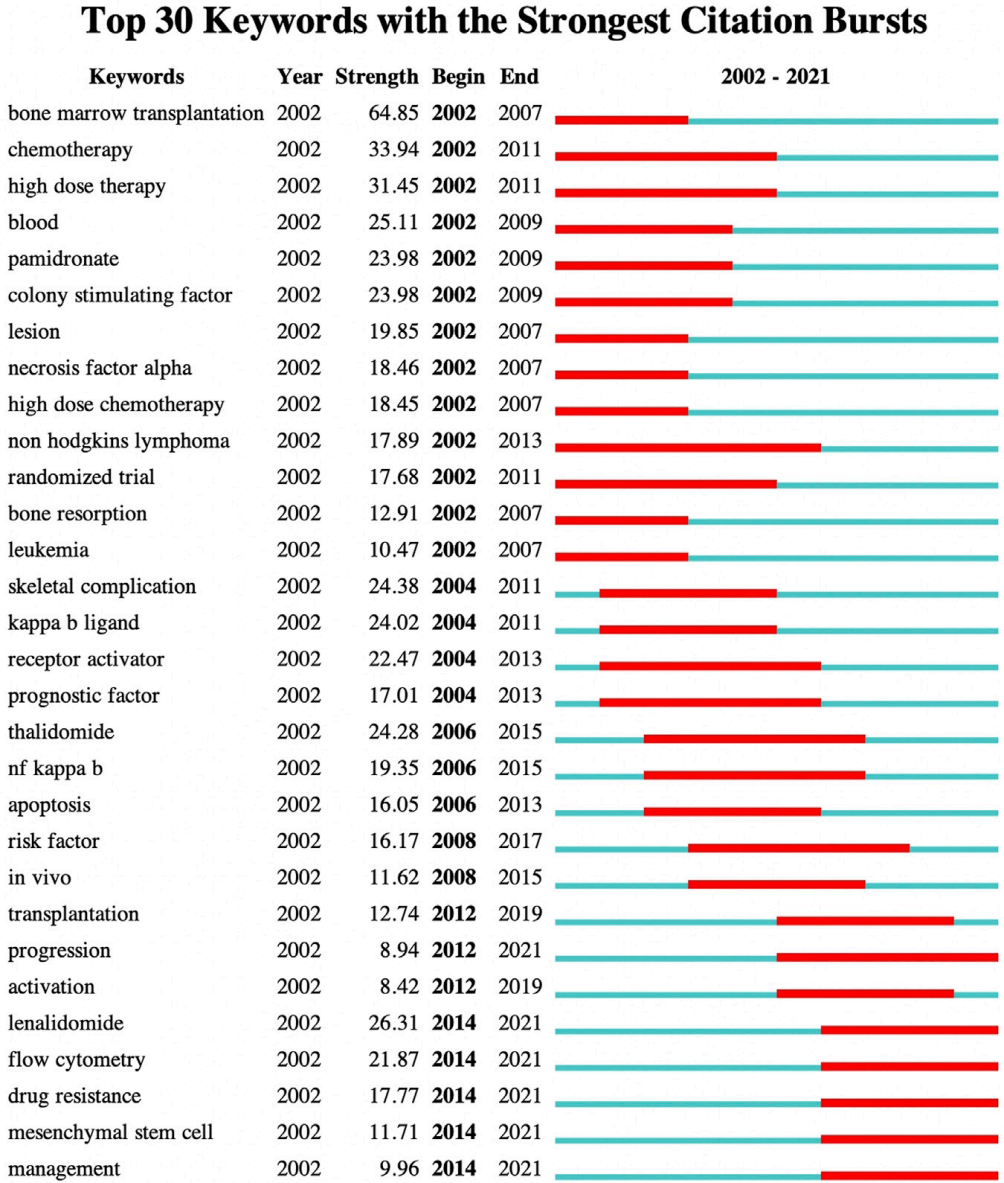
Top 30 keywords with the strongest citation bursts. The blue line indicates the year of the outbreak, and the red line indicates the time from the begin to the end of the keyword.

As the refinement and summary of the research topics and contents of the paper, keywords reflect the essence of the contents of a paper. Through the co-occurrence analysis of keywords, the research hotspots in this field can be extracted to some extent (Zhang F. et al., 2022; Miao et al., 2022; Zhang and Lin, 2022). The knowledge map of keyword co-occurrence network related to MBD consists of 138 nodes and 144 links, and Figure 9A shows the keywords with a frequency greater than or equal to 150. The top 20 keywords for frequency and betweenness centrality are listed in Table 9. Keyword clustering is based on keyword co-occurrence map using log likelihood ratio (LLR) algorithm to identify different clustering labels to determine research hotspots (Huang et al., 2021). The keyword clustering map is illustrated in Figure 9B, where clustering Q = 0.8401>0.3, S = 0.9604>0.7, indicating that the structure of the division is significant, and the clustering is highly efficient and convincing (Liu et al., 2021; Xu et al., 2022). There are 12 cluster labels in the figure, and each color block represents a cluster. The smaller the number, the larger the scale of the cluster and the more keywords involved in the cluster. Through the analysis of high frequency, high betweenness centrality keywords and keyword clustering, one finds that multiple myeloma, bone marrow transplantation, expression, stem cell transplantation, survival, therapy, zoledronic acid, monoclonal gammopathy, breast cancer, bone disease, plasma cell, diagnosis, versus host disease, bortezomib, double blind, minimal residual disease, *In vitro*, management, growth, chemotherapy, lenalidomide plus dexamethasone, phase III, high dose therapy, bone metastases, prostate cancer, progression, tumor angiogenesis, endothelial growth factor, peripheral blood, risk factor, NF-kappa B, bone marrow microenvironment and Computed Tomography (CT) are the main research topics of MBD. MM is a hematological malignancy in which plasma cells proliferate abnormally and secrete monoclonal immunoglobulin and its fragments (M protein), causing related tissue and organ damaged (van de Donk et al., 2021). MBD is caused by the interaction between malignant plasma cells and bone marrow mesenchymal stem cells, which not only increases tumor burden, angiogenesis and drug resistance, but also reduces the anti-tumor immune response of patients and affects the prognosis of MM. At present, chemotherapy, targeted therapy and stem cell transplantation for primary disease MM are the basic and most important part of the treatment of MBD, which can block or delay the pathological process of MM, and then achieve the effect of treating MBD (Charalampous and Taxiarchis, 2022; Forster and Radpour, 2022). The treatment of MBD itself includes bisphosphonates, proteasome inhibitors, immunomodulators and other drug therapy, radiotherapy and surgery are also an indispensable means (Mukkamalla and Malipeddi, 2021). Although therapeutic advances achieved in the past few years have improved prognosis and prolonged survival, MM remains largely incurable (Dutta et al., 2022; Steinmetz et al., 2022). Keyword burst refers to keywords with a surge in frequency within a short period of time. By analyzing burst keywords, the research hotspots and frontiers of the field can be judged, further reflecting the future development trend of research (Lin Z. et al., 2021; Hao et al., 2021). The top 30 keywords with the strongest citation bursts are detected by setting the minimum duration of the burst to 6 years (Figure 10). The blue line indicates the year of the outbreak, and the red line represents the time from the beginning to the end of the keyword. “Strength” is used for the burst strength. The larger the value, the higher the strength and the greater the influence of the keyword (Ai et al., 2022). Among the top 30 keywords with the strongest citation bursts, the citation burst of eight keywords ended in 2019 or later. Therefore, the eight burst keywords of transplantation, progression, activation, lenalidomide, flow cytometry, drug resistance, mesenchymal stem cell and management reflect the latest research hotspots in the field of MBD.

**TABLE 9:** The top 20 frequency and betweenness centrality of keywords related to MBD.

Rank	Keyword	Frequency	Rank	Keyword	Centrality
1	Multiple myeloma	3637	1	Bone marrow transplantation	1.20
2	Bone marrow	820	2	Chemotherapy	1.14
3	Bone marrow transplantation	705	3	Lenalidomide plus dexamethasone	1.04
4	Expression	641	4	Double blind	1.00
5	Stem cell transplantation	536	5	Phase III	0.98
6	Survival	517	6	High dose therapy	0.98
7	Therapy	450	7	Multiple myeloma	0.97
8	Zoledronic acid	416	8	Expression	0.64
9	Monoclonal gammopathy	409	9	Bone metastase	0.63
10	Breast cancer	339	10	Prostate cancer	0.62
11	Bone disease	336	11	Progression	0.35
12	Plasma cell	335	12	Tumor angiogenesis	0.30
13	Diagnosis	300	13	Zoledronic acid	0.28
14	versus host disease	238	14	Endothelial growth factor	0.28
15	Bortezomib	237	15	Stem cell transplantation	0.25
16	Double blind	235	16	Diagnosis	0.25
17	Minimal residual disease	220	17	Bone marrow	0.22
18	*In vitro*	217	18	Peripheral blood	0.20
19	Management	204	19	Risk factor	0.17
20	Growth	196	20	NF-kappa B	0.17

### Strengths and limitations

Compared with traditional literature review, bibliometric visualization analysis is more intuitive and comprehensive. To the best of our knowledge, the present work is the first comprehensive bibliometric analysis of MBD research. However, this study inevitably involves some limitations. First of all, we only searched the WoSCC database, and did not search other large medical databases such as PubMed, Scopus, or Embase, and imposed certain restrictions on language and document types, which may overlook some relevant important studies. It is worth noting, however, that WOS is the most commonly used database for bibliometrics research (Zhang M. et al., 2022; Cheng et al., 2022; Zhou et al., 2022). The purpose of restricting the research paper is to make the assessment more accurate. Secondly, as a rapidly developing field of research, the importance of some recently published high-quality studies may be underestimated due to their low citation frequency. Finally, the data generated by the papers published this year (2022) are not included in our bibliometric analysis because the database is constantly updated and this year’s dataset is incomplete.

## Conclusion

Combining the software CiteSpace and Scimago Graphica, along with the Online Analysis Platform of Literature Metrology, we have analyzed the knowledge base, research hotspots and trends of MBD publications over the past 20 years. Results show that the United States has made the greatest contribution in this field, and Mayo Clinic is the most productive institution; and that *Blood* is the most published and cited journal, marking the core journal in the field. Terpos E is found to be a major contributor in this field with considerable academic influence, and oncology and hematology are the main subject categories studied. Disease diagnosis, prognosis evaluation, pathogenesis, imaging technology and targeted therapy have become the current research hotspots in the field of MBD research. The burst keywords transplantation, progression, activation, lenalidomide, flow cytometry, drug resistance, management and mesenchymal stem cell reflect the latest research hotspots in the field of MBD. The bibliometrics and visual analysis have been employed to display the vast literature data on the knowledge map, to analyze comprehensively and systematically the knowledge framework, global trends and hotspots in the field of MBD research in an intuitive way. The development of MBD research is clarified from the analysis of research country/institution/author cooperation network, keyword co-occurrence/burst/cluster analysis and reference/author/journal co-citation network analysis, providing a valuable reference for further in-depth research to improve the diagnosis and treatment level of the MBD.

## Data Availability

The original contributions presented in the study are included in the article/Supplementary Material, further inquiries can be directed to the corresponding author.

## Supplementary material

SUPPLEMENTARY FIGURE S1 Knowledge map of co-cited journals network related to MBD. The size of the node represents the number of co-citations, the color and thickness of the node represent the number of co-citations in different periods of time, and the warmer the color is, the closer the co-citation time is. The line and its thickness indicate the co-citation and co-citation strength between journals, while the color indicates the time of the first co-citation. The nodes with purple circles represent the key nodes whose betweenness centrality is not less than 0.1.
