# Supplementary material for: Hotspots and trends in multiple myeloma bone diseases: A bibliometric visualization analysis
Source: Front Pharmacol. 2022 Oct 14;13:1003228. doi: 10.3389/fphar.2022.1003228 (PMC9614215; doi:10.3389/fphar.2022.1003228)
Supplement: Supplementary file 1 [file DataSheet1.doc]

**TABLE S1 |** The top 10 betweenness centrality of countries/ regions and institutions related to MBD

| **Rank** | **Countries/regions** | **Centrality** | **counts** | **Rank** | **Institutions** | **Centrality** | **counts** | **Countries/regions** |
| --- | --- | --- | --- | --- | --- | --- | --- | --- |
| 1 | Finland | 0.88 | 27 | 1 | Univ Washington | 0.46 | 66 | USA |
| 2 | Jordan | 0.75 | 8 | 2 | Fred Hutchinson Canc Res Ctr | 0.44 | 50 | USA |
| 3 | Saudi Arabia | 0.66 | 21 | 3 | Ohio State Univ | 0.36 | 31 | USA |
| 4 | Thailand | 0.62 | 7 | 4 | Univ Turin | 0.35 | 96 | Italy |
| 5 | Estonia | 0.60 | 3 | 5 | Hosp Univ Salamanca | 0.29 | 34 | Spain |
| 6 | Russia | 0.57 | 25 | 6 | Univ Oxford | 0.27 | 26 | England |
| 7 | Ireland | 0.44 | 40 | 7 | UCL | 0.25 | 14 | England |
| 8 | Scotland | 0.43 | 34 | 8 | Mem Sloan Kettering Canc Ctr | 0.24 | 104 | USA |
| 9 | Hungary | 0.33 | 25 | 9 | Univ Sheffield | 0.22 | 74 | England |
| 10 | Denmark | 0.31 | 104 | 10 | Univ Michigan | 0.22 | 27 | USA |

**TABLE S2 |** The top 10 co-cited journals for betweenness centrality related to MBD

| **Rank** | **Co-cited Journal** | **Centrality** | **Citation frequency** | **H-index** | **IF（2021）** | **Quartile in category** |
| --- | --- | --- | --- | --- | --- | --- |
| 1 | J Clin Oncol（United States） | 0.79 | 3540 | 494 | 50.717 | Q1 |
| 2 | Cancer Res（United States） | 0.70 | 2177 | 411 | 13.312 | Q1 |
| 3 | Leukemia（England） | 0.69 | 3341 | 176 | 12.883 | Q1 |
| 4 | Clin Cancer Res（United States） | 0.63 | 2326 | 292 | 13.801 | Q1 |
| 5 | P Natl Acad Sci USA（United States） | 0.63 | 1766 | 699 | 12.779 | Q1 |
| 6 | New Engl J Med（United States） | 0.34 | 3380 | 933 | 176.079 | Q1 |
| 7 | Blood（United States） | 0.33 | 4818 | 426 | 25.476 | Q1 |
| 8 | Cell（United States） | 0.26 | 1068 | 705 | 66.850 | Q1 |
| 9 | Oncogene（England） | 0.23 | 965 | 312 | 8.756 | Q1 |
| 10 | J Biol Chem（United States） | 0.20 | 1258 | 477 | 5.486 | Q2 |

**TABLE S3 |** The top 10 betweenness centrality of authors related to MBD

| **Rank** | **Author** | **Centrality** | **Average citation per item** | **H-index** | **Location** | **Counts** |
| --- | --- | --- | --- | --- | --- | --- |
| 1 | Dimopoulos MA | 0.36 | 73.90 | 36 | Greece | 91 |
| 2 | Sezer O | 0.31 | 160.68 | 29 | Germany | 37 |
| 3 | Richardson PG | 0.23 | 206.95 | 37 | USA | 58 |
| 4 | Hillengass J | 0.22 | 106.04 | 26 | USA | 54 |
| 5 | Rajkumar SV | 0.20 | 172.82 | 51 | USA | 89 |
| 6 | Barlogie B | 0.20 | 136.55 | 44 | USA | 77 |
| 7 | Cavo M | 0.20 | 187.14 | 34 | Italy | 42 |
| 8 | Anderson KC | 0.19 | 130.96 | 59 | USA | 123 |
| 9 | Ribatti D | 0.19 | 67.93 | 12 | Italy | 15 |
| 10 | Kyle RA | 0.18 | 148.56 | 41 | USA | 66 |

**TABLE S4 |** The top 10 frequency and centrality of subject categories related to MBD

| **Rank** | **Subject categories** | **Frequency** | **Rank** | **Subject categories** | **Centrality** |
| --- | --- | --- | --- | --- | --- |
| 1 | Oncology | 2098 | 1 | Public Environmental Occupational Health | 1.12 |
| 2 | Hematology | 1996 | 2 | Environmental Sciences Ecology | 1.09 |
| 3 | Immunology | 476 | 3 | Science Technology Other Topics | 0.93 |
| 4 | General Internal Medicine | 394 | 4 | Nanoscience Nanotechnology | 0.93 |
| 5 | Cell Biology | 342 | 5 | Cell Tissue Engineering | 0.88 |
| 6 | Research Experimental Medicine | 326 | 6 | Materials Science | 0.84 |
| 7 | Radiology Nuclear Medicine Medical Imaging | 323 | 7 | Cell Biology | 0.55 |
| 8 | Transplantation | 316 | 8 | General Internal Medicine | 0.50 |
| 9 | Pharmacology Pharmacy | 265 | 9 | Critical Care Medicine | 0.40 |
| 10 | Biochemistry Molecular Biology | 254 | 10 | Biotechnology Applied Microbiology | 0.38 |
