# Supplementary figures and images for: Hotspots and trends in multiple myeloma bone diseases: A bibliometric visualization analysis
Source: Front Pharmacol. 2022 Oct 14;13:1003228. doi: 10.3389/fphar.2022.1003228 (PMC9614215; doi:10.3389/fphar.2022.1003228)

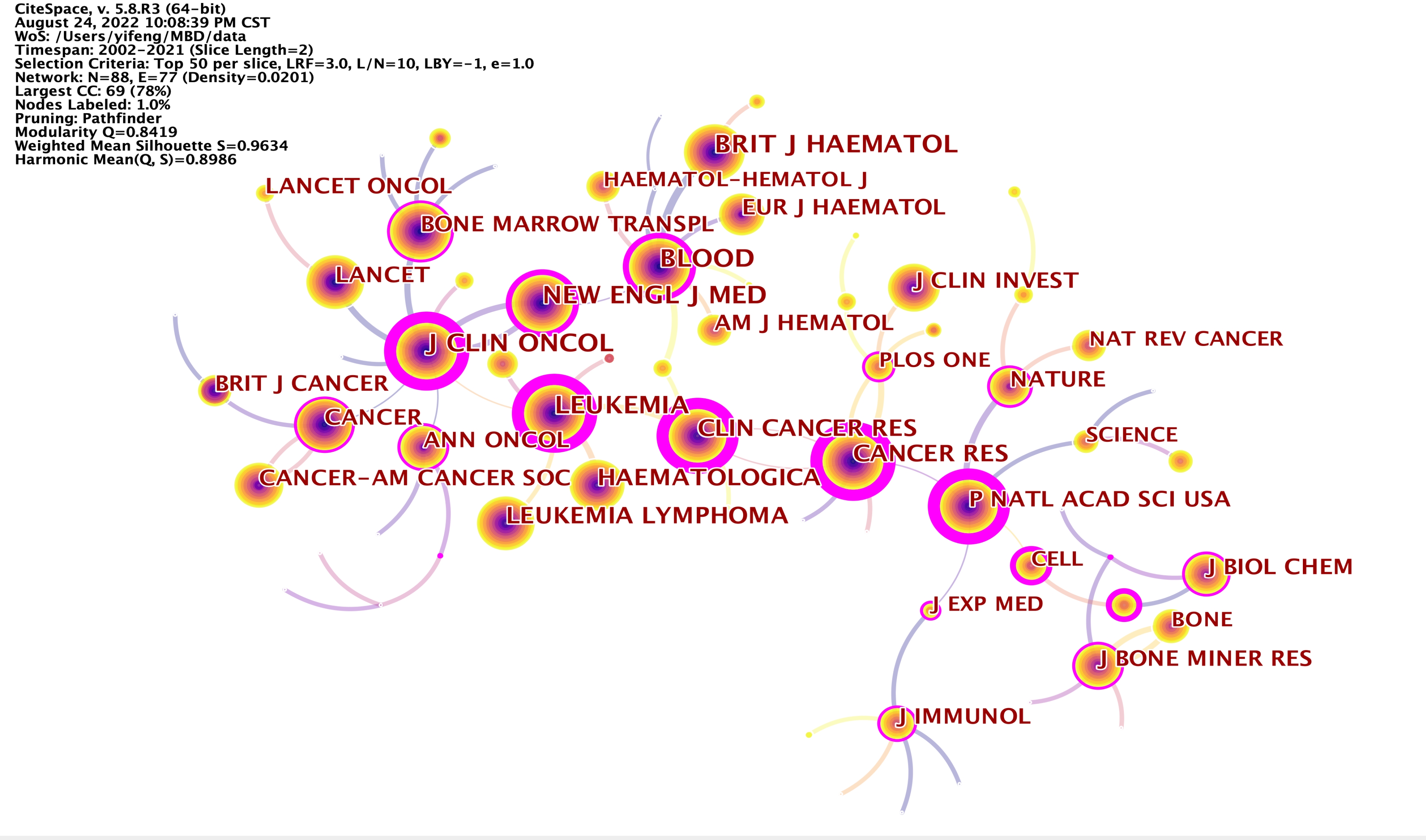

Supplement: Supplementary file 2 [file Image1.jpeg]
